# Biofluid Biomarkers of Neuronal and Glial Injury in Epilepsy and Seizures: A Comprehensive Review

**DOI:** 10.3390/ijms27198842

**Published:** 2026-10-03

**Authors:** Valentin Moroșanu, Smaranda Maier, Iulian Roman-Filip, Sergiu Moroșanu, Rodica Bălașa

**Affiliations:** 1 Doctoral School, George Emil Palade University of Medicine, Pharmacy, Science, and Technology of Targu Mures, 540142 Târgu Mures, Romania; morosanu.valentin.23@stud.umfst.ro (V.M.); iulian_roman2009@yahoo.com (I.R.-F.); rodica.balasa@umfst.ro (R.B.); 2 Neurology Department, George Emil Palade University of Medicine, Pharmacy, Science, and Technology of Targu Mures, 540142 Târgu Mures, Romania; 3 Cardiology, Targu Mures Institute for Cardiovascular Diseases and Heart Transplantation, 540136 Targu Mures, Romania; sergiu.morosanu1@gmail.com

**Keywords:** epilepsy, glial injury, neuronal injury, biomarkers, neurodegeneration, neuroinflammation, oxidative stress

## Abstract

Epilepsy is one of the most common chronic neurological disorders worldwide, affecting millions of individuals and imposing a substantial cognitive, psychological, social, and economic burden. Despite significant advances in epilepsy research, key questions remain unresolved regarding the mechanisms underlying drug resistance, cognitive impairment, depression, and sudden unexpected death in epilepsy, as well as the contribution of recurrent seizures in promoting neuronal and glial injury. Addressing these challenges requires reliable biomarkers capable of accurately reflecting disease activity and detecting ongoing brain damage. In this review, we summarize the current evidence on biofluid biomarkers of neuronal and glial damage in epilepsy and seizures and discuss their potential clinical relevance. Particular attention is given to biomarkers associated with major pathways involved in epileptogenesis, including neuroinflammation, oxidative stress, neurodegeneration, blood–brain barrier disruption, altered gene expression, and synaptic remodeling. Emerging evidence suggests that circulating and cerebrospinal fluid biomarkers may provide valuable insights into seizure-related brain injury and the molecular mechanisms underlying epilepsy. Although none of these markers has yet been adopted in routine clinical practice, they represent promising tools for improving diagnosis, prognostic assessment, patient stratification, and the development of more personalized therapeutic strategies.

## 1. Introduction

Epilepsy is one of the most prevalent chronic neurological disorders worldwide, affecting over 50 million people globally. Its impact extends beyond recurrent seizures, contributing to considerable cognitive, psychological, social, and economic challenges for patients and their families [1].

The International League Against Epilepsy (ILAE) defines epilepsy as the occurrence of at least two unprovoked or reflex seizures occurring more than 24 h apart, a single unprovoked seizure with a high risk of recurrence, or the diagnosis of a specific epileptic syndrome [2].

Despite the availability of advanced diagnostic methods, clinical assessment remains central to diagnosis, and uncertainty may persist when seizure characteristics are unclear or when episodes are not directly observed.

As reflected in the current definition, not all seizures indicate epilepsy. It is estimated that 5–10% of the population may experience a seizure at some point in their lives, and not all seizures are reflex or unprovoked. Therefore, accurately distinguishing unprovoked seizures from non-epileptic events or acute symptomatic seizures is essential for guiding treatment, predicting prognosis, avoiding unnecessary interventions, and reducing social stigma [3].

Although an estimated 5 million new cases of epilepsy are diagnosed worldwide each year, the etiology remains unknown in a substantial proportion of cases, underscoring persistent gaps in our understanding of the underlying pathophysiological mechanisms [4,5,6].

In addition to these diagnostic challenges, several aspects of epilepsy remain poorly understood. These include why some patients develop pharmacoresistant epilepsy despite the absence of detectable structural brain abnormalities, why others experience cognitive impairment, depression, or sudden unexpected death in epilepsy (SUDEP), and how recurrent seizures may contribute to neurodegeneration and progressive cognitive decline even in patients receiving appropriate antiseizure medication (ASM) therapy. Another fundamental question is whether, and under which circumstances, seizures themselves contribute to neuronal damage. Despite advances in therapeutic strategies, including epilepsy surgery, neuromodulation, and gene therapy, current treatments often fail to prevent disease onset or progression, emphasizing the urgent need for reliable biomarkers to improve understanding of disease pathophysiology, enhance diagnostic precision, and refine prognostic assessment [7,8].

The aim of this comprehensive review is to evaluate and summarize the current knowledge regarding biofluid biomarkers for detecting neuronal and glial injury in epilepsy and seizures.

## 2. Materials and Methods

This comprehensive narrative review was conducted using the PubMed database to identify relevant studies investigating biofluid biomarkers of neuronal and glial injury in epilepsy and seizures. PubMed was selected as the primary database because of its broad coverage of biomedical and clinical literature relevant to the scope of this review. A literature search was performed using combinations of terms related to epilepsy, seizures, neuronal and glial injury, and the principal biomarkers and pathophysiological pathways addressed in this review.

The core search strategy included the following combinations: “epilepsy AND biomarkers,” “seizures AND biomarkers,” “epilepsy AND neuronal injury,” “epilepsy AND glial injury,” “epilepsy AND neurodegeneration,” “epilepsy AND neuroinflammation,” “epilepsy AND oxidative stress,” and “microglia AND (epilepsy OR seizures).” Additional targeted searches were performed for individual biomarkers discussed in the manuscript, including S100B, tau, amyloid-β, ubiquitin C-terminal hydrolase L1, α-synuclein, brain-derived neurotrophic factor, glial fibrillary acidic protein, neurofilament light chain, and neuron-specific enolase, in combination with “epilepsy” or “seizures.”

The search primarily focused on studies published between 2021 and 2026 to capture recent developments in the field. The literature search was last updated in August 2026. Earlier studies were also considered when they represented seminal contributions, provided relevant mechanistic evidence, or were necessary to contextualize subsequent findings. Evidence from both adult and pediatric populations was considered. Clinical studies evaluating biomarkers in blood, cerebrospinal fluid, or other biofluids were prioritized. Experimental studies were included when they provided relevant mechanistic evidence regarding biomarker release, neuronal or glial injury, epileptogenesis, neuroinflammation, or oxidative stress. Studies were considered relevant when they investigated associations between the selected biomarkers and epilepsy, seizure activity, neuronal or glial injury, disease severity, prognosis, treatment response, or related pathophysiological mechanisms. The reference lists of relevant original articles and reviews were also screened to identify additional pertinent publications.

Given the broad, mechanistically oriented scope of this work, a narrative review approach was adopted. Accordingly, no formal meta-analysis or systematic risk-of-bias assessment was performed.

## 3. Pathophysiological Mechanisms in Epilepsy

Epileptogenesis is a complex and dynamic process associated with diverse structural, genetic, infectious, metabolic, immune, and unknown etiologies. It involves an initial precipitating event, a latent period of pathological changes, and a chronic phase characterized by recurrent seizures, with progressive neuronal and glial alterations contributing to persistent hyperexcitability and epilepsy development [9,10,11].

Several interconnected mechanisms contribute to epileptogenesis and seizure-related neuronal and glial injury. Excitotoxicity, driven by an imbalance between inhibitory and excitatory neurotransmission, may induce calcium overload, mitochondrial dysfunction, and subsequent neuronal damage [12,13,14,15].

ASMs target several of these mechanisms by modulating ion channels, enhancing inhibitory GABAergic signaling, and dampening excitatory glutamatergic activity.

Oxidative stress further promotes cellular injury through lipid peroxidation and oxidative damage to proteins and DNA [16], while neuroinflammatory responses involving activated microglia and astrocytes can alter neuronal excitability and promote reactive gliosis [17].

Synaptic reorganization and changes in gene expression contribute to maladaptive network remodeling [18,19], whereas blood–brain barrier disruption facilitates the entry of peripheral inflammatory mediators into the brain parenchyma [20].

**Figure 1:**
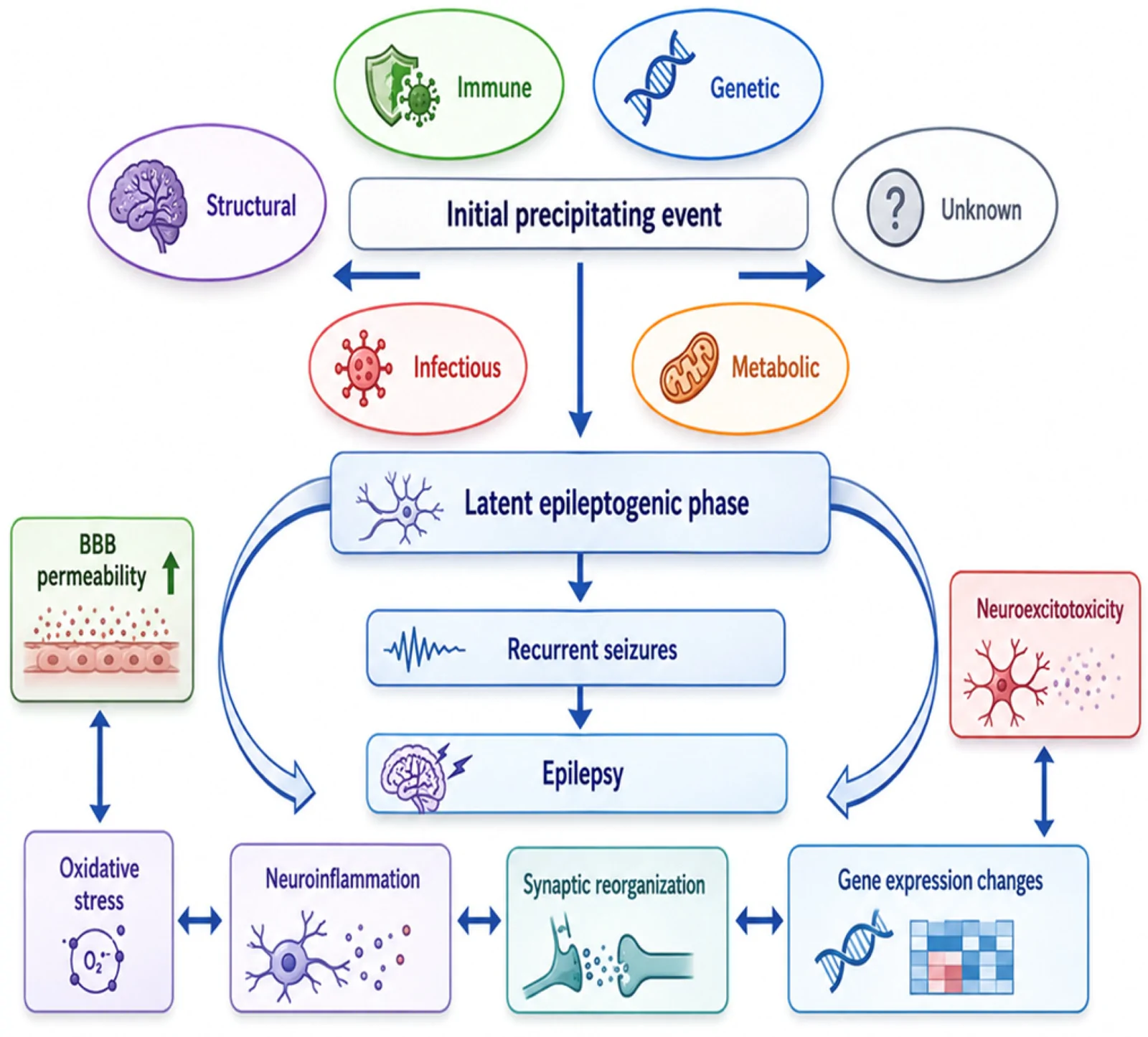
This figure illustrates the central pathological pathways involved in epileptogenesis.

Together, these processes provide the pathophysiological basis for investigating biofluid biomarkers reflecting neuronal and glial injury in epilepsy (Figure 1).

## 4. Biomarkers in Epilepsy

A biomarker is a measurable indicator of a biological or pathological process and, in some cases, may also reflect a response to a therapeutic intervention. Clinically, biomarkers are typically classified into seven categories based on their application: susceptibility, diagnostic, monitoring, prognostic, predictive, treatment-response, and safety biomarkers [21]. They can also be grouped according to the type of molecule detected (e.g., biochemical, cellular, metabolic, genetic or protein), the type of sample analyzed (e.g., blood, urine, other body fluids, tissue), and the timing of detection, which can range from the preclinical phase to established disease [22,23].

In epilepsy research, numerous biomarkers have been explored, with particular emphasis on those measurable in biofluids. Initial studies focused on markers that rise acutely following a seizure, but more recent attention has shifted toward molecules originating from the central nervous system (CNS). Although most individuals with epilepsy do not exhibit overt biochemical evidence of brain injury, ongoing research is dedicated to identifying CNS-derived molecules with potential clinical utility as biomarkers [24,25].

### 4.1. Acute Postictal Biomarkers

Epileptic seizures (ES) account for approximately 1% of emergency department visits, yet distinguishing them from non-epileptic events, particularly syncope or psychogenic non-epileptic seizures (PNES), remains a significant diagnostic challenge. Misdiagnosis can lead to inappropriate treatment, unnecessary investigations, or delays in targeted therapy, underscoring the need for objective adjunctive tools [26].

In this context, postictal serum biomarkers have gained attention as potential aids in characterizing the biochemical changes associated with ES. Among these markers, lactate measured within two hours after an event is notably elevated following generalized tonic–clonic seizures (GTCS) compared with syncope and appears to have greater diagnostic utility than creatine kinase (CK) for early differentiation [27].

In contrast, CK levels assessed 12–15 h after transient loss of consciousness are higher following GTCS than in PNES or vasovagal syncope, demonstrating moderate sensitivity and high specificity [28].

Lactate dehydrogenase (LDH) may increase within the first hour after seizure onset, while prolactin (PRL) typically peaks 10–20 min postictally, offering high specificity but variable sensitivity for ES [29,30].

Acute elevations in cortisol reflect activation of the hypothalamic–pituitary–adrenal axis and may aid in differentiating ES from PNES [31]. Serum ammonia has also shown promising discriminative performance, with a recent study reporting significantly higher levels following ES than PNES [32].

**Figure 2:**
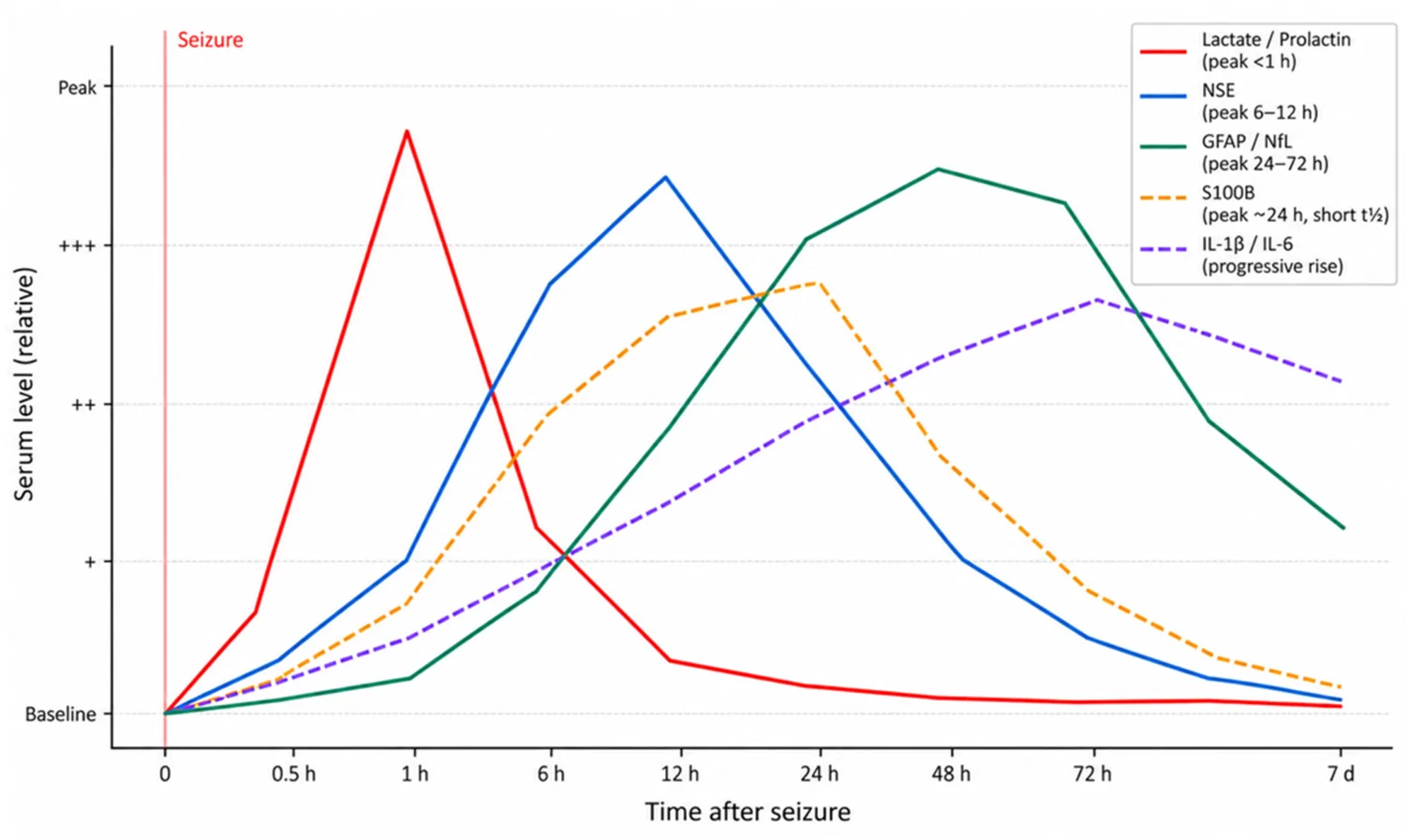
Schematic temporal profile of key postictal biomarkers.

Altogether, cortisol, lactate, CK, LDH, PRL, and ammonia represent a group of acute postictal biomarkers that may assist in the evaluation of suspected seizures by reflecting metabolic, muscular, or hormonal responses to seizure activity. However, these markers should be distinguished from biomarkers of neuronal or glial injury, as current evidence does not support their use for detecting brain tissue injury. No single postictal biomarker is definitive, and interpretation should consider the timing of sample collection, seizure subtype, and the broader clinical context. The temporal profiles of key postictal biomarkers are summarized in Figure 2.

### 4.2. Neurodegeneration Biomarkers

#### 4.2.1. S100B Protein

S100B, a calcium-binding protein predominantly expressed in astrocytes, is a widely studied biomarker of central nervous system injury, particularly in neurodegenerative diseases and post-stroke epilepsy [33].

Numerous studies, including meta-analyses, have reported elevated serum S100B levels in patients with epilepsy, although findings remain inconsistent, with some studies showing no significant differences compared with healthy controls [34,35].

Elevated S100B levels have been observed in patients with post-stroke epilepsy compared with age-matched individuals experiencing a single seizure. Similarly, adults with mesial temporal lobe epilepsy (TLE) exhibit increased serum S100B concentrations [36]. Experimental studies further support these observations. In rat models, tissue S100B concentrations correlate positively with seizure frequency and the extent of neuronal loss [37].

In pediatric populations with TLE, serum S100B levels measured within 30 min after focal seizures with impaired consciousness are significantly elevated compared with healthy controls, consistent with findings reported in adult cohorts [38].

A systematic review by Giovannini and Meletti identified S100B as a promising biomarker for quantifying neuroglial injury in status epilepticus (SE) with potential utility for improving patient prognostication and guiding neuroprotective strategies alongside antiseizure therapies [39].

Additional epidemiological evidence comes from a Danish register-based cohort study of adults with head injury, which demonstrated that serum S100B levels increased with the severity of traumatic brain injury (TBI) and were associated with a higher risk of developing post-traumatic epilepsy, particularly among patients with mild TBI who had detectable structural lesions [40].

In experimental models, seizure-inducing agents such as kainic acid and pilocarpine increase S100B levels in the hippocampus. Several compounds, including minozac, metformin, dexamethasone, and resveratrol, have been shown to lower S100B levels and improve neurological and behavioral outcomes. Similarly, inhibition of S100B production in astrocytes reduces neuroinflammation and attenuates the functional consequences of seizures. Interestingly, S100B knockout mice exhibit more rapid kindling and more severe seizures than wild-type animals, suggesting that S100B may play a context-dependent and potentially protective role in epileptogenesis [41,42].

S100B may reflect astrocyte activation and neuroglial injury following seizures, but its diagnostic value in epilepsy remains unclear. Findings across clinical studies have been inconsistent, and circulating concentrations may vary according to the underlying clinical condition and timing of sample collection. Its limited disease specificity and relatively short half-life further complicate interpretation, restricting its current use as a diagnostic biomarker in epilepsy.

#### 4.2.2. Tau Protein

Tau is a neuron-enriched microtubule-associated protein that contributes to microtubule stabilization under physiological conditions. However, abnormal hyperphosphorylation and aggregation of tau, hallmarks of tauopathies such as Alzheimer’s disease, are increasingly implicated in epilepsy and network hyperexcitability. Epileptogenesis-associated tau hyperphosphorylation may partially explain the frequent co-occurrence of tauopathy and seizures in neurodegenerative conditions, including dementia with Lewy bodies [43].

Total tau (T-tau) has been investigated primarily in cerebrospinal fluid (CSF), whereas phosphorylated tau (P-tau) accumulation has also been detected in brain tissue, particularly within the hippocampus. Both CSF and serum levels of T-tau and P-tau have been investigated as potential biomarkers in epilepsy, especially in TLE, although findings vary according to seizure etiology and clinical context [44].

Elevated CSF concentrations of total and phosphorylated tau have been reported in SE and in patients with Alzheimer’s disease who subsequently develop seizures, supporting an association between tau pathology and neuronal hyperexcitability [45]. Neuropathological studies further support this association. In a cohort of 60 young adults undergoing focal cortical resection for intractable epilepsy, tau-immunoreactive pathology was identified in 38% of cases despite the absence of diagnostic features of chronic traumatic encephalopathy.

Notably, a substantial tau burden was observed in a subset of patients, most of whom had no history of significant head trauma, and this burden did not correlate with clinical variables, suggesting unexpectedly frequent tau accumulation in a young epilepsy population [46].

Additional evidence comes from studies in neurodegenerative disease. In a cohort of 292 individuals with Alzheimer’s disease, higher CSF total tau levels and lower baseline Mini-Mental State Examination scores independently predicted the development of seizures, suggesting an association between greater underlying neurodegenerative pathology and seizure susceptibility [47].

In drug-resistant TLE, hyperphosphorylated tau pathology has been detected in up to 94% of cases in some series, with patterns overlapping Alzheimer-type and epilepsy-specific subpial distributions. Greater tau burden has been strongly associated with postoperative cognitive decline affecting verbal learning, recall, and naming, independent of most clinical risk factors, and has also been linked to a history of secondary generalized seizures [48].

However, the relationship between p-tau pathology and cognitive impairment in epilepsy remains incompletely understood. Across five studies comprising 142 patients, reported p-tau prevalence ranged widely from 3.5% to 95%, reflecting substantial methodological and pathological heterogeneity.

Limited evidence suggests that higher p-tau burden may be associated with postoperative declines in verbal learning, memory, and naming. Variability in neuropathological assessment, neuropsychological testing, and the multifactorial nature of cognitive impairment highlight the need for longitudinal whole-brain studies with standardized cognitive measures to clarify the role of tau pathology in epilepsy-related cognitive decline [49,50].

Emerging evidence also suggests bidirectional interactions between tauopathy and conditions such as TLE, post-traumatic epilepsy, and genetic epilepsies. Dysregulation of tau kinases, phosphatases, and the mechanistic target of rapamycin (mTOR) signaling pathway has been proposed to contribute to convergent or feed-forward mechanisms linking tau pathology with network hyperexcitability [51].

Experimental studies in mouse models of TLE have demonstrated seizure-associated alterations in tau expression and phosphorylation, while evidence from other experimental models suggests that tau may contribute to neuronal hyperexcitability and cognitive impairment and that reducing tau expression may mitigate seizure activity and preserve memory [52].

Nevertheless, tau abnormalities are not universally observed in epilepsy. In a recent cohort of adults with drug-resistant TLE, elevated CSF p181-tau levels were detected in only a small proportion of patients, further highlighting the heterogeneity of tau-related abnormalities in epilepsy [53]. Clarifying the interplay between tau phosphorylation, mTOR signaling, and neuronal network dysfunction may therefore reveal novel therapeutic strategies capable of targeting both seizures and epilepsy-associated cognitive decline.

Taken together, current evidence supports an association between tau pathology and selected epilepsy phenotypes, particularly TLE, but does not establish tau as a general biomarker of epilepsy-related neuronal injury. The predominance of evidence from surgical and drug-resistant cohorts further limits generalizability to the broader epilepsy population.

#### 4.2.3. Amyloid-β

Cleavage of amyloid precursor protein (APP) by β- and γ-secretases generates beta-amyloid peptides, which are involved in neurosynaptic survival and modulation of GABAergic neurotransmission under physiological conditions. Although Aβ is best known as the principal constituent of senile plaques and a pathological hallmark of Alzheimer’s disease, accumulating evidence suggests that it may also contribute to the pathogenesis of refractory epilepsy [54].

In the context of prodromal sporadic Alzheimer’s disease, CSF biomarkers alone do not reliably distinguish patients with epilepsy from those without, despite reported associations between CSF Aβ-42 and Aβ-40 levels and electroencephalographic abnormalities. These observations suggest that amyloid-β may contribute to epileptogenesis within a neurodegenerative setting. However, additional factors, including older age, hypertension, and increased white matter hyperintensities, also appear to influence seizure susceptibility, highlighting a potential vascular contribution to epilepsy in Alzheimer’s disease. Therefore, although CSF biomarkers provide valuable information regarding the pathophysiological links between Alzheimer’s pathology and epilepsy, they cannot replace electroencephalography, which remains essential for the detection of epileptiform activity [55].

Neuropathological studies further support the association between APP dysregulation and epilepsy. Sima et al. [56] investigated APP mRNA and β-APP protein expression in temporal lobe and hippocampal tissue from patients with pharmacoresistant epilepsy undergoing surgical resection. Using quantitative PCR, immunohistochemistry, and immunofluorescence techniques, the authors reported significantly increased β-APP expression in both regions, suggesting that APP dysregulation may be associated with epileptogenic brain tissue.

Epidemiological and clinical evidence also indicates a close interplay between epilepsy and neurodegeneration. Individuals with epilepsy, particularly those with late-onset epilepsy of unknown etiology, have an increased risk of developing dementia, while seizures may occur early in Alzheimer’s disease, supporting the concept of an epileptic prodrome in Alzheimer’s disease. In this context, soluble amyloid-β species may represent a shared pathological pathway linking epilepsy and cognitive decline, with Aβ-mediated epileptogenic changes occurring even before the formation of extracellular plaques [57].

Additional biomarker studies have explored seizure-related alterations in amyloid-β peptides. Shahim and colleagues analyzed CSF Aβ peptides (Aβ38, Aβ40, and Aβ42) as well as soluble APP fragments in patients following different seizure types. Although seizure-specific alterations were observed in certain Aβ isoforms, no consistent overall differences were detected compared with control subjects [58].

Similarly, a prospective observational study reported reduced CSF Aβ1–42 levels in patients with late-onset epilepsy of unknown etiology, suggesting that amyloid-related mechanisms may contribute to epileptogenesis and to the elevated dementia risk observed in this population [59].

Although evidence suggests that Aβ may contribute to epileptogenesis, its interpretation is complicated by the potential influence of underlying neurodegenerative pathology, particularly in late-onset epilepsy. Moreover, clinical studies evaluating Aβ-targeted interventions in epilepsy are lacking, leaving both its disease-specific relevance and therapeutic potential unresolved.

#### 4.2.4. Ubiquitin C-Terminal Hydrolase L1

Ubiquitin C-terminal Hydrolase L1 (UCH-L1) is a neuron-specific cytoplasmic enzyme essential for axonal integrity and neuronal survival. It plays a central role in the ubiquitin–proteasome system, a critical pathway responsible for maintaining cellular protein homeostasis through the degradation and recycling of misfolded or damaged proteins. By regulating ubiquitin turnover and facilitating the clearance of abnormal proteins, UCH-L1 contributes to the preservation of neuronal function and synaptic stability [60].

Several clinical studies investigating UCH-L1 levels in patients with epilepsy have reported significantly elevated serum concentrations compared with healthy controls, with particularly high levels observed in individuals experiencing generalized seizures and recurrent GTCS. However, no significant differences have been identified between patients during ES and those in interictal periods. In addition, CSF UCH-L1 concentrations have been shown to correlate with seizure severity and duration, suggesting a relationship with seizure-related neuronal injury [61,62].

Further evidence comes from studies conducted in emergency settings. Mondello and colleagues measured plasma and CSF levels of UCH-L1 in adults presenting to the emergency department after tonic–clonic or focal to bilateral tonic–clonic seizures and compared them with control subjects. The investigators observed significantly elevated UCH-L1 levels in both CSF and plasma within the first 12 h after seizures, supporting the potential role of UCH-L1 as an early biomarker of neuronal injury following epileptic events [63].

Animal studies suggest a neuroprotective role for UCH-L1. In a kainic acid–induced SE model, hippocampal UCH-L1 expression in the CA3 region was reduced 24 h after SE, and pharmacological inhibition of UCH-L1 prolonged seizures and exacerbated neuronal death. Similarly, in a pentylenetetrazole (PTZ)-induced rat model, co-administration of a UCH-L1 inhibitor increased seizure severity [64,65]. In contrast, in a rat model of chronic focal electrical stimulation–induced epilepsy, plasma UCH-L1 levels remained unchanged [66].

While biomarkers of neurodegeneration are well-established indicators of disease progression in adults, their relevance in pediatric populations remains less clear and, in some cases, controversial. Although some studies have reported elevated serum UCH-L1 levels in children with epilepsy, others have found no significant differences between children with refractory or non-refractory epilepsy and healthy controls. In contrast, glial biomarkers such as glial fibrillary acidic protein (GFAP) and S100B appear to be more consistently elevated in pediatric epilepsy, suggesting a potentially greater diagnostic relevance in this age group [67,68].

A cross-sectional and prospective pilot cohort study evaluating postictal serum UCH-L1 levels in patients with ES, PNES, and healthy controls reported significantly higher concentrations in ES compared with PNES and controls, although no association was observed between UCH-L1 levels and seizure type [69].

These findings suggest that UCH-L1 may be useful as a biomarker for distinguishing ES from non-epileptic events, although its value as an indicator of seizure-related neuronal injury or disease-specific cellular alterations remains uncertain. Larger prospective studies with standardized sampling intervals are therefore required before UCH-L1 can be considered for clinical implementation.

#### 4.2.5. α-Synuclein

α-synuclein is a presynaptic neuronal protein critical for neurotransmitter release, particularly in maintaining dopamine homeostasis, and for sustaining synaptic plasticity through regulation of synaptic vesicle dynamics.

Misfolded α-synuclein aggregates are well established in the pathogenesis of Parkinson’s disease, Lewy body dementia, and other synucleinopathies, although the precise mechanisms of its neurodegenerative effects remain unclear.

Beyond its recognized role in neurodegeneration, emerging evidence points to a potential, yet underexplored, involvement of α-synuclein in epilepsy [70]. Accumulation of secreted α-synuclein aggregates contributes to neurotoxicity by promoting post-translational modifications and activating astroglia, which in turn trigger oxidative stress and the release of proinflammatory cytokines [71].

Previous studies have shown that α-synuclein concentrations in serum and CSF are elevated in patients with epilepsy, particularly in those with intractable forms. In a study of 67 patients, CSF and serum α-synuclein levels were significantly higher in drug-resistant epilepsy compared with newly diagnosed or non-intractable cases, indicating a potential association with disease severity [72].

Brain tissue analysis revealed low cytoplasmic α-synuclein expression in normal brains, whereas epileptic brains exhibited markedly elevated cytoplasmic levels, which positively correlated with seizure severity during the chronic phase [73].

In patients with Alzheimer’s disease, α-synuclein levels were elevated in the amygdala of those with comorbid epilepsy or hippocampal sclerosis compared with patients with Alzheimer’s disease alone, suggesting a potential link between α-synuclein pathology and seizure susceptibility [74].

Elevated serum α-synuclein levels have been reported in pediatric epilepsy and show a positive correlation with the severity of electroencephalography (EEG) abnormalities [75].

In children with epilepsy and acquired demyelinating disorders of the CNS, serum α-synuclein levels were significantly elevated compared with healthy controls and correlated with measures of disease severity. Exosomal α-synuclein levels also strongly correlated with serum levels, supporting its potential as a noninvasive biomarker [76].

In another pediatric study, serum α-synuclein levels were significantly elevated in epilepsy patients, with the highest concentrations observed in drug-resistant cases. ROC analysis demonstrated that α-synuclein effectively distinguished epilepsy patients from healthy controls (AUC = 0.773) and drug-resistant from drug-responsive cases (AUC = 0.858). Additionally, α-synuclein levels positively correlated with seizure frequency, hospitalization rates, and the number of antiseizure medications, while showing negative correlations with time since the last seizure and age at seizure onset [77].

Experimental epilepsy models show that α-synuclein expression is upregulated in the hippocampus, with pentylenetetrazole-kindled rats and pilocarpine-induced mice exhibiting increased levels [78,79].

Although these findings suggest a possible association between α-synuclein levels and epilepsy severity, the available clinical evidence remains limited and heterogeneous. It is still unclear whether changes in α-synuclein reflect seizure-related neuronal injury or underlying neurodegenerative processes. Therefore, further longitudinal studies are needed to clarify its potential diagnostic and prognostic value in epilepsy.

#### 4.2.6. Brain-Derived Neurotrophic Factor

Brain-derived neurotrophic factor (BDNF) is one of the most widely expressed neurotrophins in the central nervous system, with particularly high expression in the hippocampus and prefrontal cortex. Because these brain regions are critical for memory, learning, emotion regulation, and executive function, BDNF has been extensively studied in several neurological and psychiatric disorders. Dysregulation or decreased levels of BDNF contribute to synaptic loss and cognitive decline in Alzheimer’s disease, behavioral symptoms in schizophrenia and depression, and appetite and energy imbalance in anorexia nervosa [80].

In epilepsy, however, the role of BDNF remains complex and context-dependent. Preclinical studies show that acute BDNF administration can induce seizures in rodents, whereas chronic exposure may reduce neuronal excitability, potentially via mechanisms such as increased central neuropeptide Y signaling [81].

Serum BDNF levels have been shown to decrease significantly following seizures, with reductions persisting from 1 to 72 h, both in individuals experiencing a single GTCS and in patients with chronic epilepsy. Notably, these alterations appear to be independent of age, epilepsy etiology, or disease duration [82].

A meta-analysis of 10 studies involving 403 patients found that overall BDNF levels were not significantly different between epilepsy patients and healthy controls; however, patients with focal epilepsy showed significantly lower BDNF levels [83].

A case–control study evaluated BDNF levels in blood serum and lacrimal fluid in patients with focal epilepsy. Compared with healthy controls, patients with epilepsy showed significantly lower BDNF levels in both serum and lacrimal fluid, although no correlation was observed between the two biological fluids. BDNF concentrations were not associated with age, epilepsy characteristics, or comorbid depression [84].

A multicenter study evaluating serum BDNF levels in patients with epilepsy found no significant associations between BDNF concentrations and epilepsy characteristics or psychiatric comorbidities such as depression and anxiety. Interestingly, the use of antiseizure medications, particularly valproate and perampanel, was associated with elevated BDNF levels [85].

Serum BDNF concentrations in 135 individuals with epilepsy did not differ significantly from those observed in healthy controls, although gender was found to influence BDNF levels. Nevertheless, reduced BDNF concentrations were associated with increased seizure frequency and longer disease duration, suggesting a negative relationship with disease severity [86].

The clinical relevance of BDNF in epilepsy remains uncertain, as reported concentrations vary across studies and may be influenced by epilepsy subtype, seizure timing, and treatment. Antiseizure medications may also affect BDNF levels, further complicating their interpretation. Therefore, BDNF cannot currently be considered a reliable diagnostic marker, although its association with disease severity deserves further investigation.

### 4.3. Glial Fibrillary Acidic Protein

GFAP is an intermediate filament protein with a relatively long half-life and is released into biofluids in response to astrocytic injury, serving as a sensitive biomarker of CNS damage and neuroinflammation.

GFAP dynamics have been extensively studied across several neurological disorders, including multiple sclerosis, Alzheimer’s disease, and Parkinson’s disease, while emerging evidence suggests that GFAP may also have potential as a biomarker in epilepsy [87,88,89,90].

Simani et al. [91] reported elevated serum GFAP levels in patients with epilepsy compared with healthy controls, particularly following recent seizures or SE.

The temporal profile of serum GFAP elevation appears to be heterogeneous across studies. Akel et al. [92] found no relationship between GFAP concentrations and the interval since the most recent seizure. In contrast, Wang et al. [93] reported a peak approximately four days after the onset of SE, with concentrations remaining elevated for up to ten days. Other studies have described persistently increased GFAP levels for several months after the event. GFAP levels also tend to increase with age, although no consistent associations have been observed with sex or disease duration [94].

Higher GFAP concentrations are generally observed in patients with more active disease, long-standing seizures, and bihemispheric involvement, and may be detected even in individuals with well-controlled epilepsy when compared to healthy controls [95].

In animal models of chemically induced SE and epileptic encephalopathies such as Dravet syndrome or SCN8A encephalopathy, GFAP is significantly upregulated in the hippocampus, dentate gyrus, and entorhinal cortex, reflecting astrocyte activation and reactive gliosis. Similarly, GFAP is elevated in Kcna1-null mice with spontaneous seizures, while levels normalize following a ketogenic diet, supporting a relationship between seizure activity, gliosis, and metabolic modulation [96,97,98,99,100].

Simani et al. [25] reported significantly higher serum GFAP concentrations following ES compared with PNES and healthy controls. In contrast, Nass and colleagues found no significant differences in GFAP levels among patients with autoimmune epilepsy, genetic generalized epilepsy, and PNES [101].

GFAP antibodies have been identified in children with unexplained focal seizures, suggesting a possible role for autoimmune astrocytopathy in pediatric epilepsy [102].

Similarly, in adult patients with autoimmune epilepsy, evidence of astrocytopathy was reflected by increased levels of GFAP autoantibodies [103].

Higher absolute GFAP concentrations have also been reported in patients with post-stroke epilepsy compared with individuals with a single seizure, although the contribution of the underlying stroke and age may complicate the interpretation of this association [104].

GFAP-δ, an isoform distinguished from other GFAP variants by its unique C-terminal domain, is selectively expressed in reactive astrocytes and has been detected in hippocampal sclerosis, as well as in balloon cells in focal cortical dysplasia and tuberous sclerosis complex lesions, while being notably absent in heterotopia. These findings suggest that GFAP-δ may serve as a valuable marker in quantifying regional gliosis in hippocampal sclerosis and for identifying balloon cells associated with tuberous sclerosis complex and focal cortical dysplasia [105]. Other GFAP splice variants, including GFAP-δ164 and GFAP-δexon6, have also been identified in focal lesions associated with chronic epilepsy, further demonstrating the heterogeneity of GFAP expression in epilepsy-associated neuropathology [106]. Experimental studies investigating peripheral GFAP in relation to seizure susceptibility have also produced variable findings, suggesting circulating GFAP may not consistently reflect susceptibility to seizure development [107].

Overall, the association between GFAP levels and seizure etiology remains limited, as elevations are often more closely related to structural brain lesions or recent seizure activity than to the underlying cause. Despite extensive evidence of GFAP alterations and astroglial activation in epilepsy, some studies have failed to demonstrate a significant association between GFAP levels and the disease itself.

### 4.4. Neurofilament Light Chain

Neurofilament light chain (NfL) is a structural axonal protein released into the CSF and bloodstream following neuroaxonal injury. It has been extensively studied in neurodegenerative disorders such as amyotrophic lateral sclerosis and Parkinson’s disease [108]. Particular attention has been given to multiple sclerosis, in which NfL has been associated with disease activity, disability progression, relapses, and MRI-detected lesions [109].

Its role in epilepsy remains incompletely understood, although ongoing research continues to clarify its involvement in the disease. A growing body of evidence suggests that serum NfL may serve as an indicator of seizure-related neuronal injury in certain epilepsy subtypes and clinical settings. Elevated NfL levels have been observed in autoimmune epilepsies, prolonged febrile seizures, SE, tonic–clonic seizures, patients with epileptogenic brain lesions detected on MRI and refractory epilepsy. Alongside GFAP, higher NfL concentrations have also been reported in patients with post-stroke epilepsy compared with individuals with a single seizure, although the underlying structural brain injury represents an important potential confounder [110,111,112].

Comparisons between patients with epilepsy and those with PNES indicate that plasma NfL, similarly to GFAP, is elevated in individuals with ES, suggesting a potential role for these biomarkers in distinguishing ES from conditions that mimic epilepsy [113].

In both convulsive and non-convulsive SE, serum NfL levels are significantly higher compared with healthy controls. The duration of the SE appears to be a major determinant of this increase. Notably, refractory and super-refractory SE, particularly episodes lasting longer than 24 h, are associated with the highest NfL concentrations. In a cohort of 204 adult epilepsy patients, 27 exhibited abnormally high NfL levels. Elevated concentrations were more common in patients younger than 65 years who had experienced seizures in the preceding two months and were associated with the presence of epileptogenic lesions on neuroimaging. Although postictal increases in serum NfL have been documented, the relationship between NfL levels and the time elapsed since the last seizure remains inconsistent across studies. In autoimmune encephalitis, such as anti-NMDA receptor encephalitis, serum NfL levels were not consistently elevated in patients with seizures, although CSF NfL concentrations increased during seizure activity or SE [114,115].

A recent systematic review by Thaele et al. identified only 19 eligible human studies from 2285 screened publications evaluating NfL and GFAP in ES and epilepsy. Although the available evidence supported their potential diagnostic and prognostic value, substantial heterogeneity was observed across patient populations, sampling times, and clinical outcomes, limiting direct comparisons between studies and the generalizability of the findings. The authors therefore emphasized the need for validation in larger and more homogeneous cohorts [116].

The clinical and pathophysiological significance of NfL in epilepsy requires careful interpretation. In adults without structural CNS lesions, serum NfL may be similar to healthy controls but can still be associated with cognitive impairment, suggesting that it reflects neuronal vulnerability rather than seizure activity. A similar observation has been reported in patients with Down syndrome, in whom NfL levels were slightly higher when epilepsy was present [117,118].

Pediatric studies indicate that significant NfL elevations are primarily observed in children with oncologic conditions or transplant rejection, rather than in typical epilepsies [119].

Current evidence suggests that NfL, alone or in combination with GFAP, may serve as a prognostic biomarker in epilepsy, particularly in cases involving structural intracerebral lesions, prolonged or severe seizures, and autoimmune-related epilepsies. However, some studies have found no significant differences in serum NfL levels between individuals with epilepsy and healthy controls, highlighting the need for cautious interpretation [120].

### 4.5. Neuron-Specific Enolase

NSE is a glycolytic isoenzyme of enolase, predominantly expressed as the γ isoform in neurons and neuroendocrine cells. Following neuronal injury or disruption of the blood–brain barrier, NSE is released into the bloodstream and cerebrospinal fluid, making it a sensitive biomarker of neuronal damage [121].

NSE has been widely studied in oncology, particularly in melanomas, seminomas, and small cell lung cancer, where its levels are associated with tumor burden, metastasis, and treatment response. It has also been investigated in a variety of neurological conditions, including Creutzfeldt-Jakob disease, Guillain-Barré syndrome, intracerebral hemorrhage, ischemic stroke, post-cardiac arrest coma, neuroendocrine tumors, and seizures [122].

According to Wen et al., elevated serum NSE levels are associated with an increased risk of epilepsy after cerebral infarction, together with factors such as severe neurological deficit, cortical involvement, and larger infarct size [123].

A meta-analysis provides robust evidence that NSE levels in both CSF and serum are significantly elevated in children with epilepsy compared to healthy controls [124].

Consistent with findings reported by Shaik et al., NSE levels are higher in critically ill neurological patients who experience seizures compared with those who do not. This observation also suggests that recurrent seizures may contribute to ongoing neuronal injury. Furthermore, DeGiorgio et al. reported significantly elevated serum NSE concentrations in patients with SE compared with controls, while Correale et al. demonstrated increased CSF NSE levels following SE [125,126].

Given that pharmacoresistance affects approximately one-third of patients with epilepsy, biomarkers such as NSE are increasingly being investigated to evaluate the effectiveness of antiseizure medication. For example, a study assessing sodium valproate, either as monotherapy or in combination with levetiracetam, in a pediatric population demonstrated greater reductions in NSE levels with combination therapy, suggesting that the combined regimen may provide a therapeutic advantage over monotherapy [127].

Antiseizure medications such as carbamazepine and oxcarbazepine have also been shown to reduce NSE levels in patients with focal seizures [128].

Changes in NSE concentrations have been associated with seizure-related neuronal injury and response to antiseizure treatment; however, their diagnostic and monitoring value in epilepsy remains unclear. Findings vary across patient populations and clinical settings, and increased NSE levels primarily indicate neuronal injury rather than epilepsy itself. This lack of disease specificity limits the current use of NSE for diagnosis, monitoring disease progression, or assessing treatment response.

### 4.6. Oxidative Stress-Related Biomarkers

Oxidative stress (OS) arises from an imbalance between the generation of reactive oxygen and nitrogen species and the brain’s antioxidant defenses, shifting redox homeostasis toward a pro-oxidative state that contributes to neuronal injury and the hypersynchronous discharges characteristic of epilepsy. Owing to its relatively limited antioxidant capacity, the brain is particularly susceptible to free radical–induced damage, which can disrupt multiple metabolic pathways, leading to lipid peroxidation, enzymatic inhibition, protein denaturation, DNA damage, and mitochondrial dysfunction [129,130].

Although the precise directionality of the relationship between OS and seizures remains incompletely understood, current evidence supports a bidirectional interaction in which OS may act both as a precipitating factor and as a downstream consequence of epileptogenesis and disease progression [131].

In patients with drug-resistant epilepsy, analysis of neocortical tissue has revealed increased reactive oxygen species (ROS) production accompanied by elevated oxidative DNA damage and altered antioxidant enzyme activity, including increased catalase (CAT), reduced glutathione peroxidase (GPx), and unchanged superoxide dismutase (SOD) levels. These findings are consistent with observations reported in animal models [132].

Plasma studies in pediatric patients with focal symptomatic or cryptogenic epilepsy similarly demonstrate dysregulated antioxidant defenses, characterized by reduced CAT activity and persistent oxidative imbalance. Refractory epilepsy has also been associated with decreased SOD, CAT, and reduced glutathione alongside elevated malondialdehyde, a marker of lipid peroxidation and systemic oxidative burden [133]. Prospective studies further indicate elevated serum GPx levels in patients with epilepsy, independent of mono- or polytherapy [134,135].

Ferroptosis, an iron-dependent form of regulated cell death characterized by the accumulation of lipid peroxides and ROS, has emerged as a potential mechanism linking oxidative damage to epileptogenesis. Experimental models demonstrate that downregulation of microRNA-211-5p (miR-211-5p) leads to upregulation of the purinergic receptor P2X7 (P2RX7), thereby enhancing neuronal excitability and seizure susceptibility. Modulation of the miR-211-5p/P2RX7 axis, either through restoration of miR-211-5p expression or pharmacological inhibition of P2RX7, attenuates seizure severity, highlighting a promising therapeutic target for mitigating ferroptosis-mediated neuronal injury [136,137].

**Figure 3:**
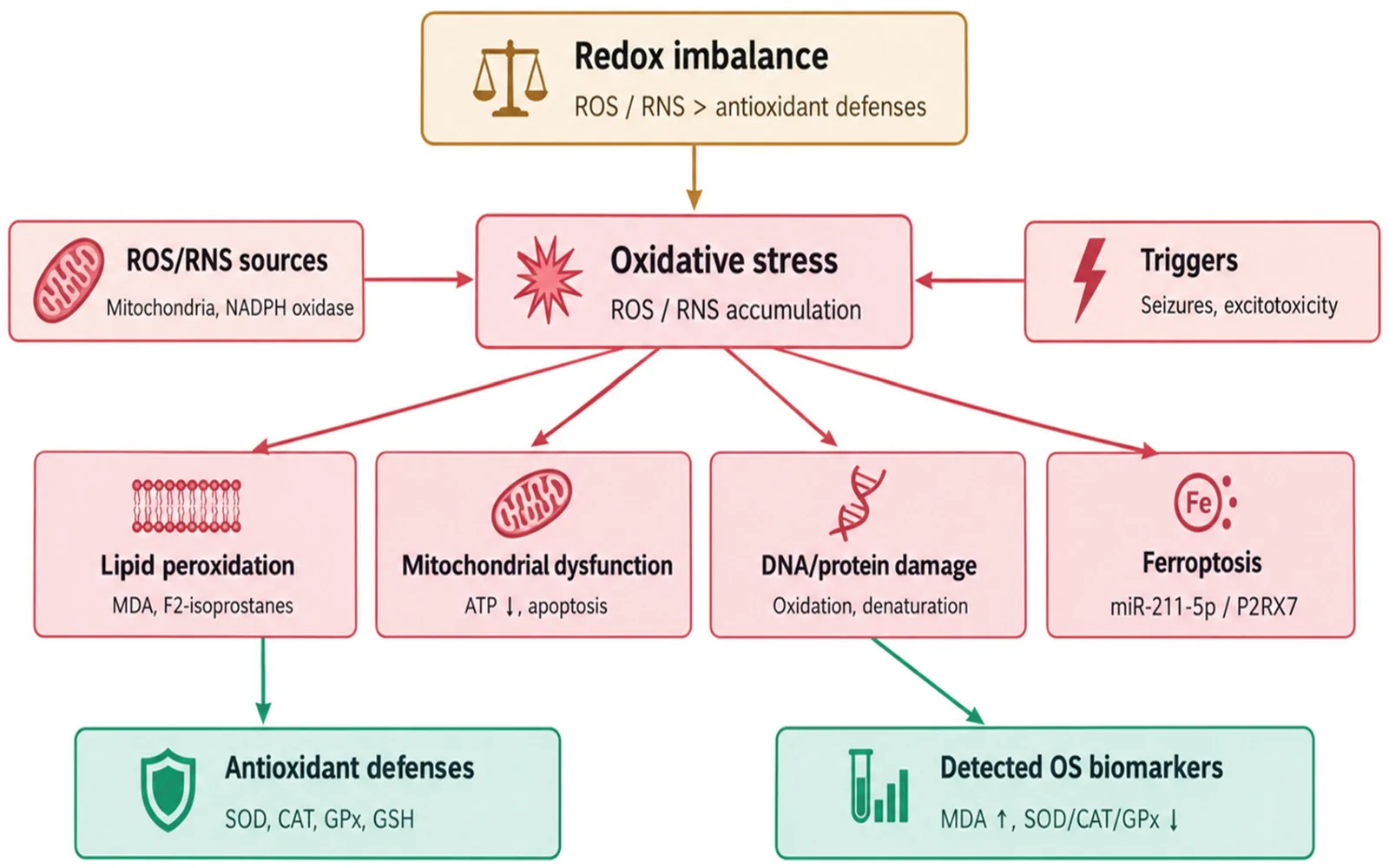
Mechanisms and consequences of oxidative stress in seizure-related neurological disorders.

Experimental evidence further supports the involvement of oxidative stress in seizure-related neuronal injury. In PTZ-, kainic acid-, and pilocarpine-induced epilepsy models, increased F2-isoprostane levels, reflecting lipid peroxidation, have been accompanied by reduced activity of endogenous antioxidant enzymes, including SOD, CAT, and GPx [138,139]. These alterations indicate disruption of redox homeostasis during seizure activity and provide a mechanistic basis for investigating oxidative stress-related molecules as potential biomarkers of neuronal injury. The main pathways involved in oxidative stress and seizure-related neuronal injury, together with the associated antioxidant mechanisms and biomarkers, are summarized in Figure 3.

Several molecular pathways involved in the regulation of oxidative stress have also been investigated. ABCG2 has been implicated in neuronal responses to oxidative injury, with experimental evidence suggesting that its activity may influence ROS-induced apoptosis and seizure susceptibility through STAT1- and mTOR-related signaling pathways [140].

Other endogenous antioxidant mechanisms, including melatonin and non-enzymatic antioxidant systems, may similarly modulate the balance between oxidative injury and cellular protection during seizures [141,142,143].

The relevance of oxidative stress to seizure-related injury is further supported by experimental studies evaluating antioxidant and neuroprotective compounds. Vitamins C and E, resveratrol, sulforaphane, curcumin, acetyl-L-carnitine, N-acetylcysteine, baicalein, coenzyme Q10, and astaxanthin have been associated with reductions in oxidative damage, seizure activity, or related neuronal injury in different experimental settings [144,145,146,147,148,149,150,151,152].

However, much of this evidence remains preclinical, and the effects reported for individual compounds cannot be directly extrapolated to human epilepsy. These findings primarily provide additional mechanistic support for the involvement of oxidative stress and impaired antioxidant defenses in seizure-related neuronal injury and for the investigation of these pathways as potential sources of biofluid biomarkers.

### 4.7. Neuroinflammatory Biomarkers

Neuroinflammation is a tightly regulated and dynamic response to endogenous and exogenous insults, characterized by activation of resident glial cells and the release of cytokines, chemokines, and other inflammatory mediators within the central nervous system. Increasing evidence implicates inflammatory signaling pathways in epileptogenesis and supports their translational relevance as candidate biomarkers [153].

Among the interleukin family, particular attention has focused on IL-1α, IL-1β, IL-33, IL-6, interferon-γ (IFN-γ), and tumor necrosis factor-α (TNF-α), with expression patterns varying according to epilepsy subtype, patient age, and seizure phase. Circulating levels of IL-1β, IL-6, TNF-α, and IFN-γ are consistently elevated following acute and recurrent seizures, underscoring their value as dynamic indicators of seizure-associated inflammatory activity [154].

IL-1β is produced predominantly by activated microglia and astrocytes. This cytokine promotes neuronal hyperexcitability by increasing glutamate release, impairing glutamate reuptake, and modulating the activity of the GluN2B subunit of the NMDA receptor. These effects impair synaptic plasticity, as evidenced by decreased long-term potentiation, which can be restored through IL-1β blockade. IL-1β also reduces GABAergic neurotransmission, further shifting the excitatory-inhibitory balance toward seizure generation [155,156].

In animal models, both IL-1β and its receptor IL-1R1 are upregulated during epileptogenesis, modulating seizure susceptibility through intracellular signaling pathways, including NF-κB and PI3K/Akt [157]. Transient postnatal IL-1β elevation following prolonged febrile seizures increases adult seizure susceptibility through IL-1R1–dependent CB1 receptor upregulation, whereas early intervention targeting this pathway prevents long-term epileptogenic outcomes [158]. Genetic variation in IL-1β and elevated CSF/serum IL-1β ratios following traumatic brain injury are associated with higher risk of post-traumatic epilepsy, emphasizing the interplay between inflammation and seizure susceptibility [159].

Clinical studies reinforce the experimental findings, demonstrating elevated IL-1β levels in brain tissue, cerebrospinal fluid, and serum of patients with drug-resistant epilepsy and febrile infection–related epilepsy syndrome (FIRES), including pediatric populations. Collectively, these data identify IL-1β as a key mediator and biomarker of neuroinflammation in epilepsy, bridging mechanistic insights from preclinical models to human disease [160].

IL-33, another member of the IL-1 cytokine family, shares structural similarities with IL-1β but exhibits distinct signaling and immunoregulatory functions. It is expressed in neurons, astrocytes, microglia, and oligodendrocytes. Elevated IL-33 correlates with increased oxidative stress in epilepsy, particularly in pharmaco-resistant cases [161].

IL-6 is a central pro-inflammatory cytokine linking neuroinflammation to epileptogenesis and disease activity. IL-6 binds to its receptor and recruits the signal-transducing subunit gp130, leading to activation of Janus kinases and phosphorylation of STAT3, thereby initiating transcriptional programs that disrupt synaptic homeostasis and promote neuronal hyperexcitability. Experimental models demonstrate that increased astrocytic IL-6 expression alters hippocampal synaptic function, contributing to network dysfunction [162,163]. Clinically, acute seizures are associated with marked elevations of IL-6 in both serum and CSF, with the highest concentrations observed following tonic–clonic seizures and generalized SE [164]. Increased IL-6 levels have been reported independently of infection markers or seizure etiology, particularly in primary generalized, secondary generalized seizures, and generalized SE [165].

Post-ictally, plasma IL-6 and IL-1 receptor antagonist (IL-1Ra) levels peak approximately 12 h after seizure onset, whereas IL-1β remains largely unchanged, suggesting selective cytokine activation [166]. Meta-analytic evidence confirms consistently elevated IL-6 levels in individuals with epilepsy compared with controls, especially in refractory epilepsy including TLE, where concentrations correlate with seizure severity and recurrence risk [167,168].

Pediatric refractory epilepsy similarly demonstrates elevated IL-6 levels, frequently accompanied by increased IL-1β and high-sensitivity C-reactive protein, consistent with a sustained pro-inflammatory state [169,170].

Interictal serum and CSF IL-6 levels correlate with seizure burden, supporting their potential utility as biomarkers of disease severity [171].

Importantly, surgical resection of epileptogenic foci reduces circulating IL-6 levels in parallel with seizure reduction. Emerging evidence also suggests that IL-6 blockade with monoclonal antibodies such as tocilizumab may reduce seizure burden in selected refractory cases [172].

TNF-α, a pro-inflammatory cytokine released predominantly by microglia and astrocytes, modulates neuronal excitability via NF-κB signaling and alters both GABAergic and glutamatergic transmission. Clinically, elevated TNF-α levels in serum and CSF have been observed in epilepsy, particularly in drug-resistant forms, and, together with IL-6, predict a higher risk of seizure recurrence [173].

Preclinical studies reveal a context-dependent role for TNF-α. While TNF-α may exert anticonvulsant effects through neuronal p75 receptors, dysregulation of p55 or p75 receptor expression appears to modify seizure outcomes [174].

In infection-induced limbic epilepsy models, elevated TNF-α and an imbalance between TNFR1 and TNFR2 enhance hippocampal hyperexcitability, partly through altered AMPA receptor trafficking [175].

Increased peripheral TNF-α levels in hippocampal sclerosis correlate with neuronal damage and reduced N-acetylaspartate concentrations. In addition, studies investigating post-traumatic epilepsy have linked increased TNF-α expression with enhanced seizure susceptibility, further supporting its role as a key mediator across multiple epileptic pathophysiological mechanisms [176,177].

#### 4.7.1. The Complement System

The complement system has emerged as a pivotal mediator of synaptic and neuronal remodeling in epilepsy. Activation of the classical pathway, initiated by C1q and followed by cleavage of C3 into iC3b, facilitates synaptic pruning during both physiological development and pathological states. In experimental models of SE, hippocampal levels of C1q, C3, and iC3b increase 2–5 weeks after SE onset, with iC3b concentrations correlating with seizure frequency. These findings suggest that sustained complement activation parallels epileptogenesis and may contribute to maladaptive synaptic remodeling [178].

Complement-mediated microglia–astrocyte crosstalk further amplifies neuronal injury. In kainic acid–induced SE models, microglia activate astrocytes through the C3–C3aR pathway, thereby exacerbating neurodegeneration. In addition, astrocyte-derived C3 may potentiate neuronal damage through TRPV1 activation, forming a C3–TRPV1–circRNA signaling axis that links glial activation to neuronal vulnerability [179,180].

Complement peptide C5a promotes seizures and neuronal hyperexcitability via C5aR1, whereas pharmacological inhibition of C5aR1 reduces both seizure burden and neuroinflammation in murine models [181].

Clinical studies further support alterations in complement activity in epilepsy. Elevated plasma levels of the terminal complement complex and factor H have been reported, and multivariate biomarker panels incorporating C3, C4, Properdin, FH, C1 inhibitor, and clusterin have demonstrated potential for distinguishing patients with SE in selected cohorts [182].

Conversely, drug-resistant epilepsy has been associated with reduced peripheral complement components, including C3 and C1q, alongside sex-specific cytokine alterations [183].

In pediatric idiopathic epilepsy, markedly decreased serum C3 levels compared with controls further support a contributory role of complement imbalance in disease pathophysiology [184].

#### 4.7.2. Matrix Metalloproteinase-9

MMP-9 is a zinc- and calcium-dependent proteolytic enzyme and a widely studied biomarker implicated in epileptogenesis. MMP-9 promotes pathological synaptic remodeling and neuronal network reorganization, thereby facilitating the formation of hyperexcitable circuits. It also amplifies neuroinflammation through activation of the receptor for advanced glycation end-products, triggering downstream cytokine release and contributing to blood–brain barrier disruption [185,186].

MMP-9 is highly enriched at excitatory synapses within the hippocampus and is upregulated by seizure activity, facilitating dendritic spine remodeling and aberrant synaptogenesis that contribute to the development of hyperexcitable neural circuits [187].

Preclinical studies support a causal role of MMP-9 in epileptogenesis. Genetic deletion of MMP-9 reduces seizure susceptibility, whereas its overexpression exacerbates epileptogenesis. In drug-resistant TLE, elevated central and peripheral MMP-9 and TIMP-1 correlate with neuroinflammatory markers, with serum MMP-9 increasing in parallel with seizure severity and decreasing after successful surgical intervention, reflecting complex central–peripheral regulation [188].

Similarly, in traumatic brain injury models, early post-injury peaks of MMP-9 contribute to the development of post-traumatic epilepsy, with MMP-9 overexpression enhancing seizure susceptibility and genetic knockout exerting protective effects [189].

Focal cortical dysplasia tissue obtained during epilepsy surgery exhibits pronounced MMP-9 upregulation in abnormal neurons, balloon cells, and synapses, indicating ongoing pathological synaptic plasticity [190].

Acute seizure-associated dynamics of MMP-9 have also been documented in humans. Serum MMP-9 transiently increases within 24 h after GTCS and returns to near-baseline levels by 72 h, supporting its involvement in blood–brain barrier disruption and seizure-related pathophysiology [191].

A meta-analysis of 13 studies (*n* = 756) confirmed significantly elevated serum MMP-9 levels in patients with epilepsy, with subgroup analysis demonstrating a significant increase in adult patients [192].

In a larval zebrafish model, transient early-life seizures induce long-term increases in seizure susceptibility and memory deficits mediated by acute post-seizure MMP-9 upregulation. Notably, brief early inhibition of MMP-9 prevented both epileptogenic and cognitive consequences [193].

Preclinical and clinical evidence supports MMP-9 as a candidate biomarker associated with seizure activity and epileptogenic processes, although its temporal variability and limited specificity constrain clinical interpretation.

#### 4.7.3. Pentraxin-3

PTX3, a member of the pentraxin family, has recently emerged as a potential mediator of immune mechanisms involved in epilepsy. PTX3 is expressed in the brain and released in response to inflammatory stimuli, where it interacts with components of the extracellular matrix and modulates AMPA receptor remodeling, thereby influencing synaptic function and neuronal circuit excitability.

Studies in experimental models, including autoimmune encephalomyelitis models, indicate that this protein is involved in the regulation of neuroinflammatory responses [194]. Its biological effects appear to be context-dependent, potentially reflecting differences in the local inflammatory environment and interactions with other cytokine-mediated pathways [195].

Evidence from a model of limbic seizures provides a more direct link with epilepsy-related pathology. Following seizure induction, PTX3 expression was rapidly increased in astrocytes and subsequently detected in neuronal populations within areas of neurodegeneration, suggesting a potential role in seizure-associated inflammation and neuronal injury [196]. The main characteristics, clinical relevance, and limitations of the biomarkers discussed in this review are summarized in Table 1.

## 5. Conclusions

Epilepsy is a heterogeneous neurological disorder whose underlying pathophysiology remains incompletely elucidated. Although multiple pathogenic mechanisms have been described, their temporal sequence and interactions remain inconsistently characterized across studies. Neuronal and glial abnormalities have been reported in epilepsy; however, it remains unclear whether these changes are causative or merely secondary to ongoing disease processes. The identification of reliable and accessible biomarkers is therefore critical for improving diagnostic accuracy, monitoring disease progression, facilitating patient stratification, and supporting the development of individualized therapeutic strategies.

Although acute postictal biomarkers may assist in distinguishing epileptic from non-epileptic events in emergency settings, they primarily reflect the systemic response to seizure activity rather than neuronal or glial injury. Their clinical utility is further limited by short half-lives and insufficient specificity.

Interest in neurodegenerative biomarkers has increased substantially, particularly among patients presenting with cognitive impairment or psychiatric comorbidities. However, these biomarkers are frequently elevated in structural and drug-resistant forms of epilepsy, as well as following prolonged or recurrent seizures, thereby complicating their diagnostic and prognostic interpretation. Their levels may also be influenced by age, epilepsy etiology, seizure type and severity, timing of sample collection, antiseizure treatment, and neurological or systemic comorbidities. Experimental studies support a relationship between seizures and increased expression of neurodegenerative markers, although the potential confounding effects of chemoconvulsant agents used in animal models must also be considered.

Neuroinflammation, oxidative stress, and epilepsy are closely interconnected processes that can influence and reinforce one another, although the sequence in which these mechanisms are initiated remains unclear.

Astroglial activation seems to play a central role in epileptogenesis through the release of pro-inflammatory mediators and reactive species. Nevertheless, therapeutic strategies focused exclusively on suppressing microglial and astrocytic activation have not yet demonstrated sufficient efficacy in preventing neuroinflammation and oxidative stress. In contrast, antioxidant and anti-inflammatory agents may reduce disease burden, suggesting that alterations in the astroglial system could, at least in part, represent secondary rather than primary phenomena. Elevated cytokine levels observed in patients with drug-resistant epilepsy are likely associated with the complexity of the morphofunctional brain alterations occurring during disease progression. Furthermore, SE, generalized seizures, and prolonged or recurrent seizures have been associated with increased concentrations of pro-inflammatory cytokines and reactive species. These findings further support the concept of bidirectional crosstalk between central and peripheral immune responses in neurological disease processes.

Despite these advances, no single biomarker currently fulfills the requirements for reliably tracking glial or neuronal injury, disease progression, or neurodegenerative risk. Nevertheless, when integrated with electroclinical and neuroimaging data, biofluid biomarkers may offer complementary insights. Continued research is essential to validate these candidates and enable their translation into clinical practice.

## Figures and Tables

**Table 1:** Summary table of neuronal and glial injury biomarkers in epilepsy.

Biomarker	Sample Type	Epilepsy Etiology	Clinical Relevance	Limitations	References
Neurodegeneration biomarkers					
S100B Origin: Astrocytes Category: Glial	Blood CSF	Structural	Elevated in post-stroke epilepsy and TLE; potential marker of neuroglial injury in SE and ES–PNES differentiation; associated with post-traumatic epilepsy risk	Short half-life; limited disease specificity; influenced by structural brain injury and seizure severity, inconsistent findings across studies	[36,39,40]
Tau Origin: Neurons Category: Neuronal	Blood CSF	Structural	Altered in TLE and SE; associated with seizure risk in Alzheimer’s disease and cognitive decline in DRE TLE	Highly variable p-tau prevalence; predominantly surgical/DRE cohorts; tau pathology not universal in epilepsy;	[44,45,47,48,49,50]
β-amyloid Origin: Neurons (APP-derived peptide) Category: Neuronal	CSF	Structural Unknown (late onset)	Reduced CSF Aβ1–42 in late-onset epilepsy of unknown etiology; altered APP expression in pharmacoresistant TLE; potential link between epilepsy and Alzheimer’s disease	Limited disease specificity; influenced by underlying neurodegeneration, age, and vascular factors; inconsistent seizure-related changes; limited interventional evidence	[54,55,56,59]
UCH-L1 Origin: Neurons Cytoplasm Category: Neuronal	Blood CSF	Unspecified	Elevated after GTCS; associated with seizure severity/duration; potential early marker of neuronal injury and ES–PNES differentiation	Inconsistent findings, particularly in pediatric epilepsy; uncertain association with seizure type and neuronal injury; sampling time-dependent; limited prospective evidence	[61,63,67,69]
α-synuclein Origin: Neurons (Presynaptic) Category: Neuronal	Blood CSF	Unspecified	Elevated particularly in DRE; associated with seizure frequency and disease severity	Limited and heterogeneous clinical evidence; unclear whether changes reflect seizure-related injury or underlying neurodegeneration	[72,74,77]
BDNF Origin: Neurons Astrocytes Category: Neuronal	Blood Lacrimal fluid	Unspecified	Reduced after seizures and in focal epilepsy; lower levels associated with higher seizure frequency and longer disease duration	Inconsistent findings; influenced by seizure timing, epilepsy subtype, sex, and ASMs; limited diagnostic specificity	[82,83,84,85,86]
GFAP Origin: Astrocytes Category: Glial	Blood CSF	Immune Structural	Elevated after recent seizures and SE; associated with structural epilepsy, including post-stroke epilepsy and malformations of cortical development; may reflect astroglial injury or activation; potential marker for ES–PNES differentiation	Age- and sampling time-dependent; influenced by structural lesions and recent seizure activity; inconsistent association with epilepsy etiology	[90,91,92,101,104,105]
NfL Origin: Neuronal axon Category: Neuronal	Blood CSF	Structural Immune	Elevated in structural epilepsy and prolonged/severe seizures; may reflect neuroaxonal injury in immune epilepsy; potential prognostic marker and ES–PNES discriminator	Limited disease specificity; reflects neuronal injury rather than epilepsy itself; elevations may occur across multiple neurological conditions	[113,114,115,116]
NSE Origin: Neurons Category: Neuronal	Blood CSF	Structural	Associated with post-stroke epilepsy risk; elevated in pediatric epilepsy and SE; potential marker of neuronal injury, changes have also been reported during antiseizure treatment	Limited disease specificity; reflects neuronal injury rather than epilepsy itself; influenced by recurrent/severe seizures and treatment	[123,124,125,127,128]
Oxidative Stress-Related Biomarkers					
SOD CAT GPx Origin: Antioxidant enzymes MDA Origin: lipid peroxidation product Category: Oxidative stress	Blood	Unspecified	Oxidative imbalance is reported particularly in DRE and focal epilepsy, oxidative pathways represent potential therapeutic targets	Variable changes across individual markers and biological compartments; limited disease specificity; predominantly associative and preclinical evidence	[132,133,134,135]
Neuroinflammatory biomarkers					
IL-1α IL-1β IL-33 IL-6 IFN-γ TNF-α Origin: Microglia, astrocytes, neurons, oligodendrocytes Category: Inflammatory	Blood CSF	Structural	Elevated following acute/recurrent seizures and in DRE; associated with seizure burden and severity; IL-1β and TNF-α implicated in post-traumatic epilepsy; potential therapeutic relevance of inflammatory pathway modulation	Limited specificity; cytokine- and context-dependent responses; influenced by seizure phase, subtype, age, and systemic inflammation	[154,159,164,167,168,169,170,171,172,177]
Complement components (C1q, C3, C4, C5a) Origin: Immune system Category: Inflammatory	Blood	Unspecified	Complement dysregulation reported in SE, DRE, and pediatric idiopathic epilepsy; associated with seizure burden, potential for distinguishing SE in selected cohorts; complement pathways represent potential therapeutic targets	Direction of changes varies across epilepsy settings; limited disease and etiology specificity; predominantly experimental evidence for mechanistic and therapeutic implications	[182,183,184]
MMP-9 Origin: Microglia, neurons Category: Inflammatory BBB-related	Blood	Structural	Elevated in DRE/TLE and after GTCS; associated with post-traumatic epileptogenesis and FCD; associated with seizure activity and epileptogenic processes	Strongly time-dependent; limited disease specificity; peripheral levels may reflect BBB and inflammatory changes	[188,189,190]
PTX3 Origin: Neurons Immune cells Category: Inflammatory	Blood	Unspecified	Potential mediator of neuroinflammatory and synaptic mechanisms in epilepsy	Predominantly preclinical evidence; relevance in human epilepsy remains uncertain	[194,195,196]
Acute postictal biomarkers					
Lactate CK LDH PRL Ammonia Cortisol Origin: Metabolic Hormonal Category: Postictal	Blood	Unspecified	Support differentiation of ES from syncope and PNES; reflect acute systemic postictal responses rather than neuronal/glial injury	Strongly time-dependent; variable sensitivity and specificity; influenced by seizure subtype and clinical context; do not indicate brain tissue injury	[26,27,28,29,30,31,32]

## Data Availability

Data supporting the findings of this study are available within the article.

## Abbreviations

Abbreviation	Full Term
AD	Alzheimer’s disease
ASM	antiseizure medication
ALCAR	acetyl-L-carnitine
APP	amyloid precursor protein
BDNF	brain-derived neurotrophic factor
CAT	catalase
CK	creatine kinase
CoQ10	coenzyme Q10
CSF	cerebrospinal fluid
DRE	drug-resistant epilepsy
ES	epileptic seizures
GFAP	glial fibrillary acidic protein
GPx	glutathione peroxidase
GSH	glutathione
GTCS	generalized tonic–clonic seizures
HS	hippocampal sclerosis
IFN-γ	interferon-γ
LDH	lactate dehydrogenase
MDA	malondialdehyde
MMP-9	matrix metalloproteinase-9
MOGAD	myelin oligodendrocyte glycoprotein antibody-associated disease
MTLE	mesial temporal lobe epilepsy
NAC	N-acetylcysteine
NfL	neurofilament light chain
NSE	neuron-specific enolase
OS	oxidative stress
PNES	psychogenic non-epileptic seizures
PRL	prolactin
PTX3	pentraxin-3
RNS	reactive nitrogen species
ROS	reactive oxygen species
SOD	superoxide dismutase
SUDEP	sudden unexpected death in epilepsy
TBI	traumatic brain injury
TLE	temporal lobe epilepsy
TNF-α	tumor necrosis factor-α
TSC	tuberous sclerosis complex
UCH-L1	ubiquitin C-terminal hydrolase L1
α-Syn	α-synuclein
